# Prognostic role of suPAR in acute pancreatitis: A protocol for systematic review

**DOI:** 10.1097/MD.0000000000037064

**Published:** 2024-06-28

**Authors:** Syeda Tayyaba Rehan, Laiba Imran, Farea Eqbal, Zayeema Khan, Abdulqadir J. Nashwan, Muhammad Sohaib Asghar

**Affiliations:** a Department of Medicine, Dow University of Health Science, Karachi, Pakistan; b Hamad Medical Corporation, Doha, Qatar; c Mayo Clinic, Rochester, MN, USA.

**Keywords:** acute pancreatitis, prognosis, soluble urokinase plasminogen activator receptor, suPAR

## Abstract

**Background:**

Acute pancreatitis (AP) is a common emergency condition with high morbidity, mortality, and socio-economic impact. Soluble urokinase plasminogen activator receptor (suPAR) is a potential biomarker for AP prognosis. This study systematically reviews the literature on suPAR’s prognostic roles in assessing AP severity, organ failure, mortality, and other pathological markers.

**Methods:**

A comprehensive search of 5 databases up to March 19, 2023, was conducted, selecting cohort studies that examined suPAR’s relationship with AP outcomes. Outcome variables included AP severity, organ failure, mortality, hospital stay length, and suPAR’s association with other inflammatory markers. Our paper has been registered on Prospero (ID: CRD42023410628).

**Results:**

Nine prospective observational studies with 1033 AP patients were included. Seven of eight studies found suPAR significantly elevated in severe acute pancreatitis (*P* < .05). Four studies showed suPAR effectively predicted organ failure risk, and 4 studies concluded suPAR significantly predicted mortality (*P* < .05). The review had no high-risk studies, enhancing credibility.

**Conclusion:**

suPAR is a valuable prognostic marker in AP, significantly predicting severity, organ failure, hospital stay length, and mortality. Further large-scale studies are needed to explore suPAR’s role in other clinical outcomes related to AP disease course, to establish it as a mainstay of AP prognosis.

## 1. Introduction

Acute pancreatitis (AP), one of the most encountered etiologies in the emergency setting, is associated with alarming levels of morbidity, mortality, and socio-economic burden.^[1]^ According to epidemiological studies the worldwide incidence of pancreatitis is on the rise with a total of 30 to 40 cases (per 100,000 population) being reported per year globally.^[2]^ The mortality of AP ranges from 3% in patients with mild edematous pancreatitis to as high as 20% in patients with pancreatic necrosis.^[3]^

**Figure 1.**
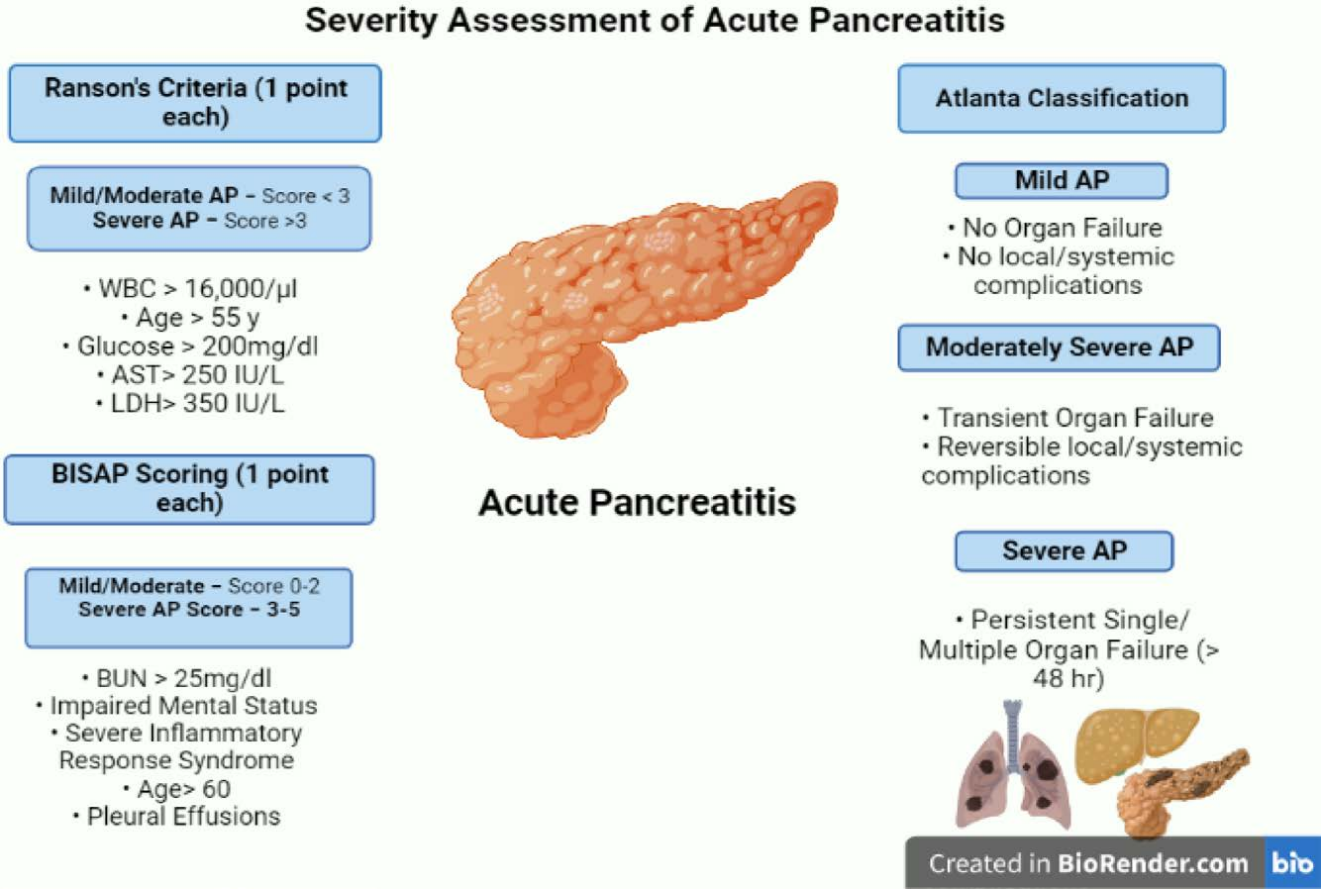
Assessment of severity in patients with acute pancreatitis by using widely established severity assessing tools, that is, Ranson criteria, Atlanta Classification and BISAP score. AP = acute pancreatitis, AST = aspartate aminotransferase, BISAP = bedside index of severity in acute pancreatitis, LDH = lactate dehydrogenase.

A vast variety of etiologies are responsible for this worrisome condition with gallbladder stones and heavy alcohol consumption being the leading factors.^[4]^ Moreover, gene mutation, hypertriglyceridemia, several drug reactions, and toxins are also implicated in the development of AP.^[5]^ Based on severity, AP can be classified as mild, moderately severe to severe acute pancreatitis (SAP; Fig. 1). Accurate assessment of the severity of AP has proven to play a pivotal role in the selection of an appropriate treatment strategy and the prediction of the clinical course of the disease, thus preventing life-threatening complications and organ dysfunction or failure.^[6]^ The prediction of the severity of AP should be achieved by a careful clinical assessment coupled with the use of a multiple factor scoring system and imaging studies.

As per the Revised Atlanta Classification 2012 (RAC), 2 morphologic subtypes of AP exist: interstitial edematous pancreatitis and necrotizing pancreatitis, while its severity has been divided into mild, moderate, and severe.^[3]^ Over the past few decades multiple scoring systems have been devised to assess the severity of AP with Ranson’s criteria and Glasgow being the most frequently employed for the purpose.^[6]^ Ranson’s scoring system is an 11-parameter score that is frequently used to evaluate the severity of acute alcoholic pancreatitis (AAP). Moreover, a modified Ranson criteria based on 10 parameters has been formulated to assess the severity of biliary AP.^[7]^ The effectiveness of this scoring system has been limited by the necessity to evaluate it at the time of hospital admission as well as 48 hours after admission, which can delay initial treatment.^[6]^ Therefore, an alternative scoring system, the Glasgow score was introduced.^[6]^ Furthermore, the Acute Physiology and Chronic Health Evaluation (APACHE II) system consisting of 12 clinical parameters,^[8]^ the bedside index of severity in acute pancreatitis (BISAP) score based on 5 factors,^[9]^ the Japanese severity score, harmless AP score, Rapid Acute Physiologic Score and Balthazar score are some of the other scoring systems employed in assessing the severity of AP.^[6]^ Figure 1 shows the severity assessing tools for AP that is, Ranson criteria, Atlanta Classification, and BISAP score.^[3,9,10]^

In addition to these scoring systems a wide range of serum markers also play a significant role in evaluating the severity of AP. Among these include Tumor necrosis factor-α, C-reactive protein (CRP), procalcitonin (PCT), Tissue factor, Hepcidin, Albumin, Total Calcium, Red blood distribution width, serum urokinase plasminogen activator receptor (suPAR), and D-dimer.^[11]^ Several investigations have proved the prognostic role of these markers in assessing the severity of AP; however, none of the current clinical scoring systems or biochemical markers have widespread applicable value or are consistently accurate.^[11]^ Therefore, early identification of the development of SAP remains a great challenge.

suPAR is emerging as a significant biomarker that has demonstrated promising results in assessing AP severity. suPAR, is a glycoprotein found in blood and other bodily fluids and is a prominent mediator of plasminogen activation and fibrinolysis.^[12]^ Since, inflammatory stimulus influences the release of suPAR from immune cells; therefore, it has been extrapolated that blood levels of suPAR illustrate inflammatory levels.^[13]^ Moreover, blood suPAR levels correlate with established inflammatory biomarkers and are minimally influenced by acute changes, enhancing its reliability in predicting severity of AP.^[11]^ Several studies have proved the importance of suPAR in predicting the emergency outcomes to aid in the prognostic stratification of patients by identifying those at high risk of deterioration.^[14,15]^

Multiple prospective observational studies^[16,17]^ have been established in the past to test the accuracy of suPAR as a prognostic marker for AP, However, no pooled analysis have been done till the date to evaluate the prognostic value of this biomarker. Considering the supremacy of suPAR in assessing AP severity coupled with the scarcity of large-scale studies centered on suPAR, it has become essential to shift the focus of research towards this novel biomarker. Therefore, in this systematic review we aim to investigate the role of suPAR in evaluating the severity, multiple organ dysfunction, mortality rates, length of hospital stay and its association with other prognostic markers in AP patients.

## 2. Methodology

### 2.1. Search strategy and data sources

This systematic review was completed in adherence with the Preferred Reporting Items for Systematic Reviews and Meta-Analyses (PRISMA) guidelines statement.^[18]^ Our paper has been registered on Prospero (ID: CRD42023410628).

A comprehensive electronic search was performed on 5 databases: PubMed (Medline), Google Scholar, Cochrane Library, ClinicalTrial.gov, and Science Direct from the inception of databases till March 19, 2023, without placing any language restrictions. An extensive search strategy was formulated by the combination of the following Medical Subject Headings (MESH terms): “AP,” “Pancreatitis,” “Alcohol induced AP,” “Acute necrotizing pancreatitis,” “Acute hemorrhagic pancreatitis,” “Soluble Urokinase Plasminogen Activator Receptor,” “suPAR.” Search strategy was adapted and modified for each database as necessary and the details of the search strategy for each database have been provided in Table S1, Supplemental Digital Content, http://links.lww.com/MD/N6. Furthermore, gray literature, website articles, books and other published and nonpublished protocols were thoroughly searched to find the additional literature.

### 2.2. Eligibility criteria

The included studies were screened based on prespecified eligibility criteria and outcome measures. We selected only those cohort studies which included participants presenting with mild, moderate, or severe AP and prospectively observed the relation of suPAR with AP outcomes. The outcome variables were defined as severity of AP, organ failure, in hospital or long-term mortality, length of hospital stay, and association of suPAR with other inflammatory markers in AP patients. Prospective observational studies without any control groups against AP patients or having healthy control individuals were eligible to be selected for our review. For severity assessment, various scoring systems were used by studies.

We excluded all the articles published in languages other than the English, nonregistered or unpublished papers, and studies having participants presenting with chronic pancreatitis (CP) or pancreatic carcinomas, or other etiologies. Furthermore, all types of reviews, case reports, case series, editorials, commentaries, and animal-based studies were also omitted from this review.

### 2.3. Data extraction

Articles yielded from the electronic search were exported to EndNote Reference Library software to remove any duplicates. Two independent reviewers (STR, FE) initially screened studies based on title and abstract and then the full text to evaluate relevance. Any discrepancies regarding the relevance of included studies were resolved by the opinion of a third reviewer (LI). The baseline demographics, study characteristics, and outcome data were extracted on an online Microsoft Excel sheet. The following baseline population and study characteristics were extracted: First author’s name, year of publication, study design, place of study, and duration of the study, number of participants, and the mean age of participants in the intervention and control groups, gender distribution, and comorbidities (Table 1).

**Table 1:** Tabulation of population characteristics.

Author (yr)	Number of participants, N	Sex	Participant characteristics	Age; Mean (SD)	Etiology of AP	
					Alcoholism n (%)	Biliary n (%)
Aronen et al^[19]^	83	*M* = 75, *F* = 8	Patients with the first AAP episode	48 (13.2)	83 (100%)	–
Long et al^[20]^	147	*M* = 81, *F* = 66	50 patients with SAP, 47 with MAP, and 50 HCs	59 (11.5)	10 (20%) (SAP)	35 (70%) (SAP)
Zhang et al^[21]^	300	*M* = 180, *F* = 120	75 patients with SAP, 75 with MSAP, and 75 with MAP	58.25 (13.5)	20 (6.6)	n = 106 (35.3)
Kolber et al^[22]^	95	*M* = 65, *F* = 30	8 patients with SAP, 58 with MSAP, and 29 with MAP.	48 (16)	–	–
Kolber et al^[23]^	95	*M* = 65, *F* = 30	Patients with AP	47.9 (16.5)	29 (30.5).	27 (28.4)
Küçükceran et al^[24]^	59	*M* = 26, *F* = 33	47 patients with MAP and 12 patients with SAP	59.64 (15)	–	40 (67.7)
Nikkola et al^[17]^	104	*M* = 85, *F* = 19	Patients with AAP	45.5 (15)	104 (100).	–
Lipinski et al^[16]^	126	*M* = 84, *F* = 42	33 patients with SAP, 37 patients with MSAP, and 56 patients with MAP	55.1 (21.9)	55 (43.3)	50 (39.4)
Friess et al^[25]^	24	*M* = 15, *F* = 9	12 patients with necrotizing AP and 12 HCs		12 (50)	–

AAP = acute alcoholic pancreatitis, AP = acute pancreatitis, HCs = Healthy controls, MAP = mild acute pancreatitis, MSAP = moderately severe acute pancreatitis, SAP = severe acute pancreatitis.

### 2.4. Quality assessment

Two reviewers (FE and LI) independently assessed the quality of the 9 included cohort studies employing the Newcastle-Ottawa scale. A third independent reviewer was consulted to resolve any discrepancies in the risk of bias assessments between the 2 review writers (STR). Newcastle-Ottawa scale acquired from http://www.ohri.ca/programs/clinical_epidemiology/oxford.asp,^[26]^ evaluates bias among studies on the basis of a “star system” in which a study is judged on 3 broad perspectives: the selection of the study groups; the comparability of the groups; and the ascertainment of the outcome of interest for cohort studies. A study can be awarded a maximum of 1 star for each numbered item within the Selection and Outcome categories and a maximum of 2 stars can be given for Comparability. A higher score indicated improved methodological quality. Studies with a total score of 8 or 9 points were considered to have a low risk of bias whereas studies with a total score of 6 or 7 points were reckoned to have a medium risk of bias. Furthermore, any score <6 points was deemed as a high risk of bias score.

### 2.5. Data synthesis

The outcome variables of recruited studies were analyzed qualitatively and were tabulated in study tables. Quantitative analysis was not possible due to methodological differences among the included studies. A *P*-value <.05 was considered as significant throughout the systematic review.

### 2.6. Literature search and study selection

**Figure 2.**
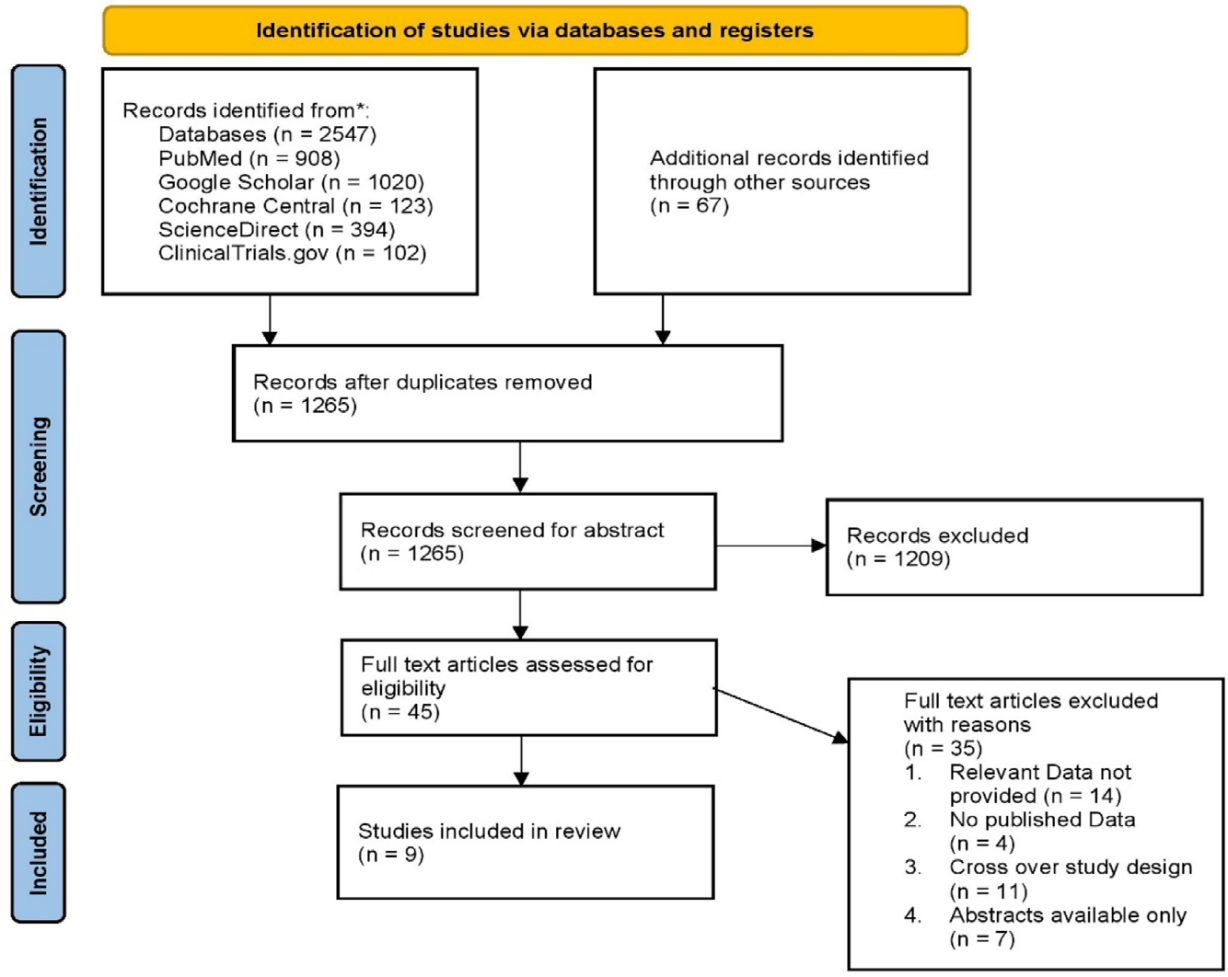
Summary of literature search.

A literature search on 5 electronic databases yielded a total of 2547 articles. After abstract screening and removing duplicates, a total of 45 full text articles were chosen for further consideration. 36 articles were then excluded as they did not meet our inclusion criteria. Finally, 9 studies have been included in our systematic review. The search and screening processes are summarized in the Preferred Reporting Items for Systematic Reviews and Meta-Analyses flowchart (Fig. 2).

### 2.7. Quality assessment

Out of a total of 9 cohort studies assessed for methodological quality, a total of 8 studies^[16,17,19, 20,22–25]^ were of medium risk and 1 study^[21]^ was of low risk. None of the included studies demonstrated high risk, enhancing the credibility of our review. The absence of a control group in most of the included studies and failure to maintain commendable follow-up data were the main factors that hindered the quality of included studies (Table S2, Supplemental Digital Content, http://links.lww.com/MD/N7).

## 3. Results

### 3.1. Study characteristics

Our review includes 9 prospective observational studies involving a total of 1033 patients.^[16,17,19–25]^ Sixty-three percentage of the patient population was male while 37% was female. All studies involved patients with AP while only 3 studies included healthy control groups.^[20,21,25]^ Out of the 1033 patients, 313 had alcoholic pancreatitis, and 258 had biliary pancreatitis whereas the etiology of pancreatitis in 462 patients was unknown. Diagnosis of AP was established based on clinical features, elevated pancreatic enzymes along with findings on Ultrasound/Magnetic Resonance Imaging.^[16,17,23,24]^ Eight studies assessed if suPAR could prove to be a useful marker for predicting the severity of AP,^[16,17,19–22,24,25]^ 4 studies evaluated its role in predicting organ failure,^[16,20,22,23]^ 5 studies evaluated the mortality rate.^[16,19–22]^ The association between suPAR and other inflammatory markers in AP was observed by five studies.^[17,20,22–24]^ A summary of the severity scoring tool for each study is presented in Table 2. A detailed description of widely used severity assessment tools and organ failure evaluation scales is presented in Table S1, Supplemental Digital Content, http://links.lww.com/MD/N6.

**Table 2:** Tabulation of study characteristics.

Author (yr)	Study design	Place of study	Duration/follow-up	Outcomes assessed	Assay used	Tools/scoring system	Findings
Aronen et sl^[19]^	Prospective observational	Finland	5 yr with a 9-yr follow-up	Severity, long-term mortality	ELISA	Atlanta classification	– suPAR level in patients with nonmild AAP was higher as compared to those with mild AAP (*P* = .047) – Patients with AAP who died during follow up had higher plasma suPAR levels compared to survivors (*P* = .008)
Long et al^[20]^	Prospective observational	China	1.5 yr	Severity, MODS, In-hospital death, relation of suPAR with other inflammatory markers	ELISA	Atlanta classification, SOFA score	– suPAR levels positively correlated with APACHE II score (*P* = .020), Balthazar index (*P* = .036), and SOFA score (*P* = .006) – Serum suPAR exhibited good value in indicating MODS – No significant correlation was found between suPAR levels and in-hospital death (*P* = .451) – Serum suPAR demonstrated a positive association with serum D-dimer level (*P* = .285) and serum PCT level (*P* = .104), but without statistical significance
Zhang et al^[21]^	Prospective observational	China	2 yr	Severity, in-hospital mortality	ELISA	Atlanta classification	– suPAR levels were elevated in SAP patients compared to MSAP patients (*P* = .23), MAP patients (*P* < .001), and healthy controls (*P* < .001) – suPAR was increased in in-hospital deaths compared to patients with AP who survived (*P* < .001)
Kolber et al^[22]^	Prospective observational	Poland	1.5 yr	Severity, organ failure, in-hospital mortality, association with inflammatory markers	ECLIA	Atlanta classification, MMSS	– uPAR levels were higher in patients with SAP as compared to MSAP and MAP (*P* > .05) – Serum suPAR concentration was a significant indicator of organ failure, acute kidney injury, acute cardiovascular failure, and ICU transfer (*P* < .05) – suPAR levels were a significant predictor of in-hospital death (*P* < .05) – uPAR concentrations were linked with procalcitonin, IL-6, and CRP. Albumin and hematocrit were adversely linked with serum uPAR
Kolber et al^[23]^	Prospective observational	Poland	1.5 yr	MOF, association with inflammatory markers	ECLIA	MMSS	– uPAR concentration was significantly higher in patients who had organ failure (MMSS ≥ 2 points; *P* < .05) – NLR levels were highly associated with serum uPAR whereas a statistically significant adverse correlation was found between LMR and uPAR (*P* < .05)
Küçükceran et al^[24]^	Prospective observational	Turkey	–	Severity, association with inflammatory markers	ELISA	Ranson score, Glasgow (Imrie), RAPS, and Balthazar’s scores	– suPAR levels were significantly elevated in the patient group with SAP (*P* < .001) – White blood cell count, neutrophil count, glucose, urea, creatinine, LDH, AST, ALT, and direct bilirubin were all increased in patients who had SAP along with elevated suPAR (*P* < .05)
Nikkola et al^[17]^	Prospective observational	Finland	5 yr	Severity, association with inflammatory markers	ELISA	Atlanta classification	– suPAR levels were significantly higher in nonmild AAP when compared to mild AAP (*P* < .001) – Compared to CRP, creatinine, and hematocrit, p-suPAR concentration of ≥5.0 ng/mL was the only independent predictor of AAP severity (OR = 21.1)
Lipinski et al^[16]^	Prospective observational	Poland	–	Severity, organ failure, mortality, length of hospital stays	Double monoclonal antibody sandwich enzyme immunoassay	BISAP score of≥3, MMSS	– Serum suPAR concentration in patients with SAP, was higher as compared to MSAP and MAP patients (*P* < .0001) – Increased serum suPAR levels were associated with an increased risk of progression to MOF with a cutoff of 5.20 ng/mL – High suPAR concentration appeared to predict fatal AP with the cutoff value of 7.05 ng/mL – suPAR levels were significantly associated with the length of hospital stay (*P* < .001)
Friess et al^[25]^	Prospective observational	Switzerland and Germany	–	Severity	–	Immunohistochemistry and northern blot analysis.	– uPAR levels were elevated in the pancreatic parenchyma adjacent to the necrosis as compared to parenchyma that has less morphological destruction

AAP = acute alcoholic pancreatitis, ALT = alanine aminotransferase (ALT), AST = aspartate aminotransferase, AP = acute pancreatitis, APACHE II = Acute Physiology and Chronic Health Evaluation II, BISAP = bedside index of severity in acute pancreatitis, CRP = C-reactive protein, ECLIA = electrochemiluminescence immunoassay, ELISA = enzyme-linked immunosorbent assay, LDH= lactate dehydrogenase, LMR = lymphocyte–monocyte ratio, MAP = mild acute pancreatitis, MMSS = Modified Marshall Scoring System, MODS = multiple organ dysfunction syndrome, MOF = multiple organ failure, MSAP = moderately severe acute pancreatitis, NLR = neutrophil- lymphocyte ratio, PCT = procalcitonin, RAPS = Rapid Acute Physiologic Score, SAP = severe acute pancreatitis, SOFA = Sequential Organ Failure Assessment, suPAR = soluble urokinase-type plasminogen activator receptor, uPAR = urokinase-type plasminogen activator receptor.

### 3.2. Clinical outcomes

#### 3.2.1. Severity of AP

A total of 8 included studies observed the role of suPAR in evaluating the severity of AP.^[16,17,19–22,24,25]^ The severity was further classified as mild acute pancreatitis (MAP), moderately severe acute pancreatitis (MSAP), and severe acute pancreatitis (SAP) based on different preestablished classification systems. Four studies made use of the Atlanta classification for grading the severity of AP^[17,19,21,24]^ while 1 study employed the use of a BISAP score of ≥3 for the diagnosis of SAP.^[16]^ Küçükceran et al^[24]^ classified the severity of AP based on the Ranson score, Glasgow (Imrie), RAPS, and Balthazar scores.

Seven of the eight studies reported that suPAR was significantly elevated in SAP as compared to MAP^[16,17,19–21,24,25]^ whereas 1 study found no significance of suPAR in predicting the severity of AP.^[22]^ Lipinski et al^[16]^ determined the cutoff point for serum suPAR concentration in differentiating between SAP and MSAP/MAP to be 4.75 ng/mL with a sensitivity and specificity of 97% and 93% respectively. Serum suPAR concentration in patients with SAP, MSAP, and MAP was found to be 8.8 ± 4.9, 3.8 ± 0.8, and 2.6 ± 0.7 ng/mL respectively (*P* < .0001).^[16]^ Nikkola et al’s^[17]^ study found that the suPAR levels were significantly higher in nonmild AAP (6.2 ng/mL, 5.0–7.9) when compared to mild AAP (4.2 ng/mL, 3.1–5.6; *P* < .001). The optimal cutoff level for nonmild AAP was 5.0 ng/mL, with sensitivity and specificity being 79% and 78% respectively.^[17]^ Zhang et al^[21]^ reported that suPAR levels were elevated in SAP patients compared to MSAP patients (*P* = .23), MAP patients (*P* < .001), and healthy controls (*P* < .001). Results from Kucukseran et al’s^[24]^ study found that suPAR levels were significantly elevated in the patient group with SAP (*P* < .001), with the optimum cutoff value for determining the severity of AP being 6.815 ng/mL. Aronen et al’s^[19]^ study performed a nine year follow up of patients with AP. Plasma suPAR level in patients with nonmild AAP was higher for 12 months as compared to those with mild AAP (*P* = .047). Kolber et al’s^[22]^ study, however, showed that uPAR levels, while higher in patients with SAP as compared to MSAP and MAP, were not statistically significant. Friess’ et al’s^[25]^ study described the cellular distribution and expression of uPAR in acute necrotizing pancreatitis (ANP) using immunohistochemistry and northern blot analysis. Results showed increased expression of uPAR in ANP samples as compared to healthy controls. Moreover, uPAR levels were elevated in the pancreatic parenchyma adjacent to the necrosis as compared to the parenchyma that has less morphological destruction.^[25]^

Three studies compared the suPAR levels with other severity scales in predicting AP severity.^[19,21,24]^ Long et al^[20]^ reported that suPAR levels positively correlated with APACHE II score (*P* = .020), Balthazar index (*P* = .036), and Sequential Organ Failure Assessment (SOFA) score (*P* = .006). However, there was no positive association between serum suPAR levels and Ranson score (*P* = .207).^[20]^ This contrasts with the studies conducted by Zhang et al^[21]^ and Kolber et al^[22]^ which showed a positive correlation between suPAR levels and Ranson score (*P* < .001 and *P* = .012 respectively).

#### 3.2.2. Organ failure

Four studies assessed the role of suPAR in predicting organ failure in AP patients.^[16,20,22,23]^ Three studies utilized the Modified Marshall Scoring System (MMSS)^[16,22,23]^ to diagnose organ failure while 1 study employed the use of SOFA score.^[20]^ All 4 studies demonstrated that serum suPAR was effective in predicting the risk of developing organ failure in AP.^[16,20,22,23]^ Lipinski et al^[16]^ reported that increased serum suPAR levels were associated with an increased risk of progression to multiple organ failure. The optimal cutoff for suPAR levels was 5.20 ng/mL with a sensitivity of 93% and specificity of 90%.^[16]^ Kolber et al^[22]^ reported that serum suPAR concentration was a significant indicator of organ failure, acute kidney injury, acute cardiovascular failure, and Intensive Care Unit transfer (*P* < .05). Long et al^[20]^ reported serum suPAR exhibited good value (area under the curve [AUC]: 0.782; 95% CI, 0.656–0.907) in indicating multiple organ dysfunction syndrome (MODS), with a sensitivity and specificity of 72.4% and 76.2%, respectively. Serum suPAR was better at predicting MODS when compared with Balthazar, showed a similar value when compared to Ranson, and was inferior to APACHE II and SOFA in predicting MODS.^[20]^ In another study, Kolber et al^[23]^ reported that uPAR concentration was significantly higher in patients who had organ failure (MMSS ≥ 2 points) as compared to those who did not present with organ failure (*P* ≤ .05).

#### 3.2.3. Mortality

The role of suPAR in predicting the mortality rate was evaluated by 5 studies.^[16,19–22]^ Mortality was either assessed as in-hospital mortality^[20–22]^ or long-term mortality.^[16,19]^ Four studies concluded that suPAR could significantly predict mortality in patients with AP^[16,19,21,22]^ while 1 study reported no such significant association.^[20]^ Lipinski et al^[16]^ reported that the concentration of suPAR was an indicator of mortality in AP with 7.05 ng/mL being the optimal cutoff point, demonstrating a sensitivity of 83% and specificity of 90%. Aronen et al^[19]^ reported that patients with AAP who died during follow up had higher plasma suPAR levels compared to survivors (*P* = .008). The cutoff value for suPAR was 3.4 ng/mL with a sensitivity of 58% and specificity of 82%. The 10-year mortality risk was 13% and 49% when suPAR was <3.4 or ≥3.4 ng/mL, respectively.^[19]^ According to the results of Zhang et al’s^[21]^ study, suPAR increased in-hospital deaths compared to patients with AP who survived (*P* < .001). Moreover, suPAR’s value in indicating hospital mortality was like the Ranson score, APACHE II score, SOFA score, and CRP.^[21]^ Kolber et al’s^[22]^ study reported that suPAR levels were a significant predictor of Intensive Care Unit transfer and hospital death (*P* < .05). In contrast, Long et al^[20]^ reported that AP patients who died in the hospital had higher levels of suPAR as compared to survivors, but the difference was not statistically significant (*P* = .451).

#### 3.2.4. Association of suPAR with other inflammatory markers

Five studies observed the association of suPAR with other inflammatory markers in AP.^[17,20,22–24]^ Nikkola et al^[17]^ reported that when compared to other laboratory markers such as CRP, creatinine, and hematocrit, a p-suPAR concentration of ≥5.0 ng/mL was the only independent predictor of AAP severity. According to Kucukseran et al^[24]^, laboratory parameters such as white blood cell count, neutrophil count, glucose, urea, creatinine, lactate dehydrogenase (LDH), aspartate aminotransferase (AST), alanine aminotransferase (ALT), and direct bilirubin were all increased in patients who had SAP, with the exception of creatinine (*P* = .016, *P* = .006, *P* = .048, *P* = .011, *P* = .022, *P* = .004, *P* < .001, *P* = .027, and *P* = .039, respectively). Similarly, in the same study, suPAR levels were also significantly elevated in patients with SAP as compared to MAP (*P* < .001), showing a positive correlation between suPAR and other laboratory parameters used to assess the severity of AP.^[24]^ Kolber et al^[22]^ reported that uPAR concentrations were linked with various inflammatory indicators during the study period, including procalcitonin, IL-6, and CRP (beginning on day 2 of the hospital stay). On admission, there was a link between uPAR and sFlt-1, and on days 1 and 3, there was a correlation between uPAR and D-dimer.^[22]^ Albumin and hematocrit were adversely linked with serum uPAR throughout the trial.^[22]^ Moreover, uPAR showed positive correlations with LDH, aminotransferases, and bilirubin.^[22]^ There was no connection between uPAR and the levels of serum urea or creatinine.^[22]^ Findings from Long et al’s^[20]^ study show that serum suPAR demonstrated a positive association with serum D-dimer level (*P* = .285) and serum PCT level (*P* = .104), but without statistical significance. Kolber et al^[23]^ reported that throughout their study, neutrophil–lymphocyte ratio (NLR) levels were highly associated with serum uPAR whereas a statistically significant adverse correlation was found between lymphocyte–monocyte ratio and uPAR on days 1 and 2 (*P* < .05).

## 4. Discussion

AP is the chief cause of hospital admissions for gastrointestinal disorders in the US and countries across the globe.^[27]^ It serves as the second leading cause of overall hospital stays, the fifth leading cause of in-hospital mortality, and has the largest contribution to overall hospital costs.^[27]^ Statistics reveal that it is imperative to deal with AP patients before the disease proceeds to severity and results in multiple systemic complications. To predict the severity and progress of AP, different prognostic scoring systems have been devised and implemented for decades. This includes the BISAP score, APACHE II,^[28]^ Ranson and Glasgow score.^[6]^ However, none of the currently used scoring systems are accurate in indicating the severity of the disease in the early stages and are also inconvenient to use in patients.^[6]^

Considering the drawbacks of the scoring systems currently utilized in AP, it is essential to explore new laboratory markers or tools that can not only predict severity but can also indicate other consequences of AP such as organ failure and mortality. suPAR has emerged as an efficient method in indicating the prognosis in patients with multiple inflammatory conditions including AP and could help decrease the significant mortality and morbidity that is associated with the poor prognostic tracking of the disease. suPAR is the soluble form of urokinase-type plasminogen activator receptor (uPAR) and is positively correlated with inflammation and immune activation.^[29]^ Research has come to light that shows that suPAR may play a major role in predicting outcomes of AP such as severity, organ failure, and fatality.^[16,17,19–25]^

Our systematic review pools the results of 9 prospective observational studies investigating the role of suPAR as a prognostic marker in AP.^[16,17,19–25]^ From the results of our review, we observed that suPAR can be used as a prognostic marker for indicating AP severity, as 8 out of the 9 studies showed that suPAR levels were significantly higher in patients who had SAP as compared to mild cases of AP.^[16,17,19–22,24,25]^ Previous meta-analyses have shown the undeniable role of suPAR as an important biomarker in various diseases such as kidney disease,^[29]^ sepsis,^[30]^ and cancer.^[31]^ Recent studies also demonstrate how suPAR acts as an inflammatory mediator and prognostic marker in cardiovascular disease,^[32]^ systemic lupus Erythematosus (SLE),^[33]^ and Covid-19.^[34]^ suPAR was significantly increased in patients with COVID-19 and acute kidney injury and turned out to be an excellent indicator of disease severity and outcome.^[29,34]^ suPAR also proved to be valuable in differentiating the severity of the disease in patients with SLE (*P* < .001) and had a diagnostic sensitivity and specificity of 100%.^[33]^ suPAR’s role in assessing disease severity also extends to chronic hepatitis B infection as research shows that suPAR was able to differentiate between severe and mild liver fibrosis with adequate specificity and sensitivity (*P* < .05).^[35]^

Given that indicator like CRP, the standard for diagnosing organ failure,^[36]^ are ineffective during the first 48 hours after disease onset,^[6]^ suPAR has the potential to be an alternative predictor of organ failure. All studies that assessed the role of suPAR in predicting organ failure in AP reported it as an effective biomarker for evaluating the risk of progression to organ failure.^[16,20,22,23]^ Persistent organ failure during the first week in patients presenting with AP is associated with a 1 in 3 risk of mortality.^[37]^ Currently, markers such as urea, partial pressure of oxygen and hemoglobin are used to predict mortality in AP, however, no single prognostic marker has been established as the gold standard, probing research into markers that can accurately predict mortality.^[38]^

Our study summarizes suPAR as a significant marker to predict short-term and long-term mortality and length of hospital stay in AP.^[16,19,21,22]^ A previously conducted meta-analysis has proved suPAR’s role in moderately assessing mortality in sepsis.^[30]^ According to a meta-analysis conducted by Li et al^[39]^, suPAR can also be used as a biomarker to indicate all cause and cardiovascular mortality. Enocsson et al’s^[40]^ study evaluating the relationship between suPAR levels and COVID-19 found that suPAR concentration was positively correlated with the length of hospital stay (*P* = .006).

Plasma and serum suPAR levels have been established to be positively associated with conventional markers of inflammation such as CRP, erythrocyte sedimentation rate, procalcitonin, fibrinogen, neutrophils, monocytes, red blood cell count, white blood cell count, and various cytokines and chemokines in patients with systemic chronic inflammation.^[41,42]^ In our review, we assessed if any such correlation existed between suPAR and other inflammatory markers in AP. In comparison to CRP, creatinine, and hematocrit, suPAR emerged as the only independent indicator of AP severity.^[17]^ Moreover, serum suPAR was found to be elevated in patients with SAP along with other laboratory parameters such as glucose, urea, and creatinine indicating a positive correlation between the 2.^[24]^

suPAR’s role as an invaluable prognostic biomarker in AP is evident from the results of our review. However, there is currently a paucity of literature that explores this relationship and more large-scale studies need to be conducted before suPAR can be established as a mainstay of prognosis in AP. We also urge that more research is done that investigates the role of suPAR in CP as a study report that suPAR can also aid in differentiating between CP and pancreatic cancer (*P* = .009).^[43]^ Considering that the morbidity and mortality surrounding pancreatitis is high, suPAR could be the prognostic breakthrough that helps improve outcomes in patients.

### 4.1. Strengths and limitations

To the best of our knowledge, this is the first systematic review that pools the role of suPAR as a prognostic marker in AP. Out of the 9 studies selected for this review, none of them showed a high risk of bias, further adding to the strength of our paper. However, our review has certain limitations. Firstly, all the included studies are prospective observational as no clinical trials have been performed that investigate suPAR’s role in AP. The optimal cutoff for suPAR was different across all the studies, hence no threshold could be decided that gives an accurate predicting value for suPAR. Secondly, the sample size of the respective studies is small and more large-scale research needs to be conducted to reach concrete results. Lastly, the data present on this topic was not adequate to carry out a meta-analysis hence no analysis was run.

## 5. Conclusion

suPAR is a valuable prognostic marker in AP and can significantly predict outcomes such as severity, organ failure, length of hospital stays, and mortality. Most of the current literature only reports suPAR’s efficacy in assessing disease severity in AP. More large-scale studies are required to explore suPAR’s role in relation with other clinical outcomes that are associated with the course of the disease so it can be established as a mainstay of prognosis in AP.

## Author contributions

**Conceptualization:** Syeda Tayyaba Rehan, Laiba Imran, Farea Eqbal, Zayeema Khan, Abdulqadir J. Nashwan, Muhammad Sohaib Asghar.

**Data curation:** Laiba Imran, Farea Eqbal, Zayeema Khan, Abdulqadir J. Nashwan, Muhammad Sohaib Asghar.

**Formal analysis:** Laiba Imran.

**Investigation:** Zayeema Khan.

**Methodology:** Farea Eqbal.

**Project administration:** Syeda Tayyaba Rehan, Laiba Imran, Farea Eqbal, Zayeema Khan, Abdulqadir J. Nashwan, Muhammad Sohaib Asghar.

**Resources:** Abdulqadir J. Nashwan.

**Software:** Farea Eqbal, Zayeema Khan.

**Supervision:** Muhammad Sohaib Asghar.

**Writing – original draft:** Syeda Tayyaba Rehan, Laiba Imran, Farea Eqbal, Zayeema Khan, Abdulqadir J. Nashwan, Muhammad Sohaib Asghar.

**Writing – review & editing:** Muhammad Sohaib Asghar.

## Supplementary Material




