# Supplementary material for: Prognostic role of suPAR in acute pancreatitis: A protocol for systematic review
Source: Medicine (Baltimore). 2024 Jun 28;103(26):e37064. doi: 10.1097/MD.0000000000037064 (PMC11466201; doi:10.1097/MD.0000000000037064)
Supplement: Supplementary file 1 [file medi-103-e37064-s001.docx]

**Supplementary Table 1: Search strategy used in each database searched.**

| **Database** | **Search Strategy** | **Articles retrieved** |
| --- | --- | --- |
| PubMed | "pancreatitis"[MeSH Terms] OR "pancreatitis"[All Fields] OR ("acute"[All Fields] AND "pancreatitis"[All Fields]) OR "acute pancreatitis"[All Fields] OR (("alcohol s"[All Fields] OR "alcoholate"[All Fields] OR "alcoholates"[All Fields] OR "alcoholic s"[All Fields] OR "alcoholics"[MeSH Terms] OR "alcoholics"[All Fields] OR "alcoholic"[All Fields] OR "alcoholism"[MeSH Terms] OR "alcoholism"[All Fields] OR "alcoholisms"[All Fields] OR "alcoholism s"[All Fields] OR "alcoholization"[All Fields] OR "alcohols"[MeSH Terms] OR "alcohols"[All Fields] OR "ethanol"[MeSH Terms] OR "ethanol"[All Fields] OR "alcohol"[All Fields]) AND ("induce"[All Fields] OR "induced"[All Fields] OR "inducer"[All Fields] OR "inducers"[All Fields] OR "induces"[All Fields] OR "inducibilities"[All Fields] OR "inducibility"[All Fields] OR "inducible"[All Fields] OR "inducing"[All Fields]) AND ("pancreatitis"[MeSH Terms] OR "pancreatitis"[All Fields] OR ("acute"[All Fields] AND "pancreatitis"[All Fields]) OR "acute pancreatitis"[All Fields])) OR ("acute necrotising pancreatitis"[All Fields] OR "pancreatitis, acute necrotizing"[MeSH Terms] OR ("pancreatitis"[All Fields] AND "acute"[All Fields] AND "necrotizing"[All Fields]) OR "acute necrotizing pancreatitis"[All Fields] OR ("acute"[All Fields] AND "necrotizing"[All Fields] AND "pancreatitis"[All Fields])) OR ("pancreatitis, acute hemorrhagic"[MeSH Terms] OR ("pancreatitis"[All Fields] AND "acute"[All Fields] AND "hemorrhagic"[All Fields]) OR "acute hemorrhagic pancreatitis"[All Fields] OR ("acute"[All Fields] AND "hemorrhagic"[All Fields] AND "pancreatitis"[All Fields])) AND (("solubility"[MeSH Terms] OR "solubility"[All Fields] OR "solubilities"[All Fields] OR "soluble"[All Fields] OR "solubles"[All Fields] OR "solublization"[All Fields] OR "solublize"[All Fields] OR "solublized"[All Fields]) AND ("receptors, urokinase plasminogen activator"[MeSH Terms] OR ("receptors"[All Fields] AND "urokinase"[All Fields] AND "plasminogen"[All Fields] AND "activator"[All Fields]) OR "urokinase plasminogen activator receptors"[All Fields] OR ("urokinase"[All Fields] AND "plasminogen"[All Fields] AND "activator"[All Fields] AND "receptor"[All Fields]) OR "urokinase plasminogen activator receptor"[All Fields])) OR "suPAR"[All Fields] | 908 |
| Google Scholar | (Soluble Urokinase Plasminogen Activator Receptor OR Urokinase plasminogen activator receptors OR Receptor urokinase plasminogen activator OR suPAR OR pancreatitis OR Acute pancreatitis OR Alcohol induced acute pancreatitis OR Acute necrotizing pancreatitis) | 1020 |
| Cochrane Library | (Soluble Urokinase Plasminogen Activator Receptor OR Urokinase plasminogen activator receptors OR Receptor urokinase plasminogen activator OR suPAR) | 123 |
| Clinicaltrials.gov | (Soluble Urokinase Plasminogen Activator Receptor OR Urokinase plasminogen activator receptors OR Receptor urokinase plasminogen activator OR suPAR) | 102 |
| Science Direct | (Acute pancreatitis OR Alcohol induced acute pancreatitis OR Acute necrotizing pancreatitis OR Acute hemorrhagic pancreatitis) AND (Soluble Urokinase Plasminogen Activator Receptor OR suPAR) | 394 |
