# Supplementary material for: Prognostic role of suPAR in acute pancreatitis: A protocol for systematic review
Source: Medicine (Baltimore). 2024 Jun 28;103(26):e37064. doi: 10.1097/MD.0000000000037064 (PMC11466201; doi:10.1097/MD.0000000000037064)
Supplement: Supplementary file 2 [file medi-103-e37064-s002.docx]

**Supplementary Table 2: Newcastle - Ottawa Quality Assessment Scale for cohort studies:**

| **Study** | **Selection** | | | | **Comparability** | **Outcome** | | | **Total^a^** |
| --- | --- | --- | --- | --- | --- | --- | --- | --- | --- |
|  | **1** | **2** | **3** | **4** | **5** | **6** | **7** | **8** |  |
| Lipinski (2016) [15] | * | _ | * | * | ** | * | * | _ | 7 |
| Nikkola (2017) [16] | * | _ | * | * | ** | * | * | * | 7 |
| Zhang (2019) [19] | * | * | * | * | ** | * | * | * | 8 |
| KÜÇÜKCERAN (2018) [20] | * | _ | * | * | ** | * | * | _ | 6 |
| Aronen (2019) [21] | * | _ | * | * | ** | * | * | * | 7 |
| Kolber (2018) [22] | * | _ | * | * | ** | * | * | _ | 6 |
| Long (2019) [23] | * | _ | * | * | ** | * | * | _ | 6 |
| Kolber (2018) [24] | * | _ | * | * | ** | * | * | _ | 6 |
| Friess (1998) [25] | * | * | * | * | ** | * | * | _ | 7 |

**a= out of a maximum score of 9**

**Scoring systems in acute pancreatitis**

**Atlanta classification**

This revised classification of acute pancreatitis identified two phases of the disease: early and late. Severity is classified as mild, moderate or severe. Mild acute pancreatitis, the most common form, has no organ failure, local or systemic complications and usually resolves in the first week. Moderately severe acute pancreatitis is defined by the presence of transient organ failure, local complications or exacerbation of co-morbid disease. Severe acute pancreatitis is defined by persistent organ failure, that is, organ failure >48 h.

**Bedside Index for Severity in Acute Pancreatitis (BISAP)**

In 2008, the Bedside Index for Severity in Acute Pancreatitis (BISAP) score was proposed for the early recognition of patients at risk of mortality. This 5-point scoring system is comprised of five variables: blood urea nitrogen level > 25 mg/dl, impaired mental status, development of systemic inflammatory response syndrome (SIRS), age > 60 years, and presence of pleural effusion.

**Ranson criteria**

The original Ranson criteria is a scoring system that uses 11 parameters to assess the severity of acute pancreatitis. The 11 parameters are age, white blood cell count (WBC), blood glucose, serum aspartate transaminase (AST), serum lactate dehydrogenase (LDH), serum calcium, fall in hematocrit, arterial oxygen (PaO2), blood urea nitrogen (BUN), base deficit, and sequestration of fluids.

**Glasgow Imrie score**

It is alternative clinical scoring system for both alcohol and biliary acute pancreatitis. Eight laboratory factors are needed within the first 48 h of treatment to calculate it and more than three positive criteria indicate severe acute pancreatitis.

**Rapid Acute Physiology Score (RAPS)**

The Rapid Acute Physiology Score (RAPS) was developed and tested for use as a severity scale in critical care transports. RAPS is an abbreviated version of the Acute Physiology and Chronic Health Evaluation (APACHE-II) using only parameters routinely available on all transported patients (i.e. pulse, blood pressure, respiratory rate, and Glasgow Coma Scale). RAPS has a range from 0 (normal) to 16.

**Balthazar score**

The Balthazar CT severity index (CTSI) is one of the reliable severity grading systems applied to human medicine. This grading system evaluates acute necrotizing pancreatitis based on the morphology of the pancreatic parenchyma and necrosis as depicted by CT imaging.

**Modified Marshal Scoring System (MMSS)**

The original Marshall Score has been developed to provide an objective measure of ICU mortality in patients with various clinical presentations, taking into consideration six organ systems, central nervous system (CNS), hepatic, respiratory, cardiovascular, renal and hematologic systems, giving each system a certain score depending on its degree of dysfunction. A modified, simpler version of this score has been later adopted to stratify AP patients into mild, moderate and severe presentation, which correlated with their mortality.

**The sepsis-related organ failure assessment (SOFA)**

The SOFA score was created to describe multiple organ failure in critically ill patients and to allow the monitoring of therapies. It individualizes the degree of pulmonary, coagulation, hepatic, renal, central nervous system and cardiovascular dysfunction daily.
